# Optimally Repeatable Kinetic Model Variant for Myocardial Blood Flow Measurements with ^82^Rb PET

**DOI:** 10.1155/2017/6810626

**Published:** 2017-02-13

**Authors:** Adrian F. Ocneanu, Robert A. deKemp, Jennifer M. Renaud, Andy Adler, Rob S. B. Beanlands, Ran Klein

**Affiliations:** ^1^Systems and Computer Engineering, Carleton University, Ottawa, ON, Canada; ^2^National Cardiac PET Centre, Division of Cardiology, Department of Medicine, University of Ottawa Heart Institute, Ottawa, ON, Canada; ^3^Department of Nuclear Medicine, The Ottawa Hospital, Ottawa, ON, Canada; ^4^Division of Nuclear Medicine, Department of Medicine, University of Ottawa, Ottawa, ON, Canada

## Abstract

*Purpose.* Myocardial blood flow (MBF) quantification with ^82^Rb positron emission tomography (PET) is gaining clinical adoption, but improvements in precision are desired. This study aims to identify analysis variants producing the most repeatable MBF measures.* Methods.* 12 volunteers underwent same-day test-retest rest and dipyridamole stress imaging with dynamic ^82^Rb PET, from which MBF was quantified using 1-tissue-compartment kinetic model variants: (1) blood-pool versus uptake region sampled input function (Blood/Uptake-ROI), (2) dual spillover correction (SOC-On/Off), (3) right blood correction (RBC-On/Off), (4) arterial blood transit delay (Delay-On/Off), and (5) distribution volume (DV) constraint (Global/Regional-DV). Repeatability of MBF, stress/rest myocardial flow reserve (MFR), and stress/rest MBF difference (ΔMBF) was assessed using nonparametric reproducibility coefficients (RPC_np_ = 1.45 × interquartile range).* Results.* MBF using SOC-On, RVBC-Off, Blood-ROI, Global-DV, and Delay-Off was most repeatable for combined rest and stress: RPC_np_ = 0.21 mL/min/g (15.8%). Corresponding MFR and ΔMBF RPC_np_ were 0.42 (20.2%) and 0.24 mL/min/g (23.5%). MBF repeatability improved with SOC-On at stress (*p* < 0.001) and tended to improve with RBC-Off at both rest and stress (*p* < 0.08). DV and ROI did not significantly influence repeatability. The Delay-On model was overdetermined and did not reliably converge.* Conclusion.* MBF and MFR test-retest repeatability were the best with dual spillover correction, left atrium blood input function, and global DV.

## 1. Introduction

Repeatable myocardium blood flow (MBF) measurements are essential to detect minute changes in myocardial perfusion due to disease progression or in response to therapy, as well as for accurate clinical classification in comparison to population databases. Quantification of MBF requires a series of image analysis steps including the use of a tracer kinetic model and accurate correction for partial-volume losses (and corresponding signal mixing effects) [1]. Various models have been investigated in the literature [2], but most commonly a 1-tissue-compartment model (also known as the 2-compartment model) with a tissue-blood volume estimation is used to describe the kinetics of ^82^Rb [3]. Models are often simplified to improve model stability and robustness in the presence of image noise; however, this is potentially at the expense of physiological or physical completeness.

Various factors influence repeatability. Schindler et al. [4] and Efseaff et al. [5] evaluated elements of image reconstruction. Klein et al. [6] evaluated tracer infusion. DeKemp et al. [7] and Bravo et al. [8] looked at the agreement in software implementation. Moody et al. [9] looked at the effects of the tracer extraction function on MBF variance. In this work, we focused on reducing the variability introduced by the 1-tissue-compartment kinetic model, by comparing previously described variants of it, thus attempting to identify the most repeatable kinetic model variant.

Previous work by our team evaluated MBF repeatability using rest imaging alone and reported a test-retest repeatability coefficient (95% limits of agreement) as low as 20% [5] using optimal image reconstruction parameters. These results were confirmed using both cardiac-rest and cardiac-stress imaging in a recent study by our group that concluded better repeatability using constant-activity (versus constant-flow) infusion of ^82^Rb tracer and using a 1-tissue-compartment model (versus a simplified retention model) [6]. The present study expands on these previous studies to determine most precise 1-tissue-compartment model variants. Thus, our goal in this study was twofold: (1) to elucidate the effects of more physically and physiologically complete kinetic model variants on MBF repeatability and (2) to identify the 1-tissue kinetic model variant which achieves the most precise MBF quantification. In this work, we describe alternative variants of the 1-tissue-compartment model and compare their repeatability for quantifying MBF and MFR with ^82^Rb PET using a single imaging session, test-retest study.

## 2. Materials and Methods

### 2.1. Patient Recruitment and Preparation

This study reanalyzed the constant-activity (CA) cohort data previously reported in [6]. Study participants were instructed to abstain from caffeine intake for 12 hours, fast for 4 hours (except for water intake), and withhold medication according to standard clinical guidelines prior to the exam [10]. Clinical demographics, cardiac risk factors, and history of cardiac procedures and medications were recorded for each subject. Patients with acute coronary syndrome or unstable angina, heart failure, pulmonary edema, severe valve disease, or contraindication to dipyridamole such as hypotension, heart block, or asthma were excluded. All participants provided written informed consent. The study was approved by the Human Research Ethics Board at the University of Ottawa Heart Institute (UOHI). Due to technical reasons that are explained later, one patient was excluded from the study.

### 2.2. Image Acquisition Protocol

**Figure 1:**
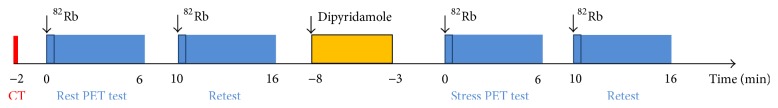
Rest and stress imaging protocol.

A modified clinical protocol [11], as illustrated in Figure 1, was used to acquire two rest and two stress scans in a single session, in an attempt to maintain consistent patient positioning and hemodynamic conditions between test and retest. Patients were positioned in a Discovery 690 PET/VCT scanner (GE Healthcare, Waukesha, WI) with ECG leads placed for patient monitoring and cardiac gating. Patient heart rates, blood pressure, and symptoms of ischemia were monitored throughout the imaging session.

A scout scan was performed for patient positioning, followed by a low-dose (<0.3 mSv) normal end-expiration breath-hold CT for attenuation correction. Four PET list-mode scans were acquired, each following 10 MBq/kg of ^82^Rb administered as a constant-activity [12] “square-wave” infusion over a 30-second interval using a RubyFill-V2 infuser (Jubilant DraxImage, Kirkland, QC). This was done to limit the scanner coincidence dead-time to <35% for accurate measurement of the bolus first-pass activity, while ensuring sufficient counts to achieve high quality uptake and ECG-gated images for routine myocardial perfusion imaging (MPI) interpretation [6, 13]. All scans were initiated manually after ^82^Rb infusion was started and the PET scanner reported that coincidence count rates exceeded 10 kcps.

The first rest scan (test) was followed immediately by a second (retest). The stress agent, dipyridamole (0.14 mg/kg/min), was infused for 5 minutes, and 3 minutes later two stress image acquisition procedures (test + retest) were performed in quick succession, as shown in Figure 1. Four minutes after starting the second stress scan, aminophylline was administered to the patient to reduce symptoms.

### 2.3. Image Reconstruction

PET images were manually adjusted, if necessary, for optimal registration with the CT data to ensure accurate attenuation correction. List-mode scan data were binned into 14 time frames (10 s × 9, 30 s × 3, 60 s × 1, 120 s × 1) and iteratively reconstructed using the vendor OSEM method (VuePointHD, 24 subsets, 4 iterations) with an 8 mm 3D Hann postfilter and were corrected for physical decay of the tracer [5].

### 2.4. Image Analysis

All images were processed using in-house custom MBF quantification software, FlowQuant v.2.4 (UOHI, Ottawa, ON), by a single operator to ensure consistent image orientation and segmentation (see SM 1). Late uptake-phase (2–6 min) images were automatically reoriented, with optional operator intervention to produce short-axis (SA) slices through the left ventricle (LV). The LV myocardium was then segmented into 496 individual sectors in which the arterial time-activity curves (TACs) were sampled [11]. Ventricular and atrial cavity regions of interest (ROIs) were also segmented to generate arterial TACs as detailed in the Arterial Input ROI. All the other processing was completely automated and was therefore free of any potential operator bias. All model variants were based on the commonly used 1-tissue-compartment model [11], in which the tracer activity in the myocardial tissue is modeled as

(1) CttK1e−k2t⊗Cbloodt=K1e−K1/DVt⊗Cbloodt,

where *C*_*t*_(*t*) is the modeled time-dependent myocardial tissue activity concentration, *K*_1_ is the ^82^Rb uptake rate in mL/min/g, *k*_2_ is the ^82^Rb rate of washout from the myocardium in min^−1^, *C*_blood_(*t*) is the image-derived tracer concentration in arterial blood, and ⊗ is the discrete convolution operator. DV is the distribution volume, the ratio of tissue, and blood tracer concentrations after the compartments reach equilibrium and can be expressed as

(2) $$\begin{array}{c} DV=\frac{K_{1}}{k_{2}}. \end{array}$$

For each polar map sector, the myocardial image concentration was modeled as

(3) $$\begin{array}{c} C_{myo}\left(\;t\;\right)=FBV\times C_{blood}\left(\;t\;\right)+\left(\;1-FBV\;\right)\times C_{t}\left(\;t\;\right), \end{array}$$

where FBV represents the fractional blood volume (unitless) and (1 − FBV) was used to correct for regional recovery of partial-volume losses in the myocardium [14]. The *K*_1_ and FBV parameters were estimated using (1) and (3) for each sector of the LV myocardium, via weighted (by frame length) nonlinear least squares minimization of differences between the modeled and the sampled myocardium TACs, *C*_myo_(*t*).

An extraction correction function *E*(MBF), as defined by Lortie et al. [15], was used to convert *K*_1_ values to MBF:

(4) $$\begin{array}{c} K_{1}=\left(\;1-{0.77e}^{-0.63/MBF}\;\right)MBF. \end{array}$$

Variants of the model are described in the next 5 sections.

#### 2.4.1. Distribution Volume

We explored two variants of the distribution volume: (1) with a free DV parameter (Regional-DV) and (2) with a spatially uniform DV constant (Global-DV) that was determined by fitting the free model to the normal uptake region within the polar map (>75% maximum) [5]. By using a constant value for DV, the number of model parameters is reduced, thereby increasing the model optimization robustness, potentially at the expense of regional accuracy.

#### 2.4.2. Spillover Correction (SOC)

The image-derived arterial input function, *C*_blood_(*t*), may be contaminated by spillover signal from the myocardium into the LV cavity due to the limited image spatial resolution. A practical dual-spillover correction technique [5] was used to derive a pure blood signal *C*_*b*_(*t*) that replaces *C*_blood_(*t*) as the input function to the kinetic model. The image-sampled TACs for blood and whole-LV average, *C*_blood_(*t*) and *C*_myo_wholeLV_(*t*), respectively, were assumed to consist of a complementary mix of pure blood, *C*_*b*_(*t*), and pure myocardial tissue signals, *C*_*t*_(*t*), such that

(5) Cmyo_wholeLVt=FBV×Cbt+1−FBV×Ct_wholeLVt,Cbloodt=β×Cbt+1−β×Ct_wholeLVt,

where *β* is the fraction of pure blood signal in image-sampled blood TAC (typically in the order of 85% [16]). Thus, the pure blood signal, *C*_*b*_(*t*), can be derived from (5), as a function of *C*_blood_(*t*) and *C*_myo_wholeLV_(*t*), using

(6) Cbt=1−FBV×Cbloodt−1−β×Cmyo_wholeLVtβ×1−FBV−1−β×FBV.

In this approach, (1) and (5) were used to approximate *K*_1_, *k*_2_, FBV, and *β* based on the average whole-LV values of *C*_myo_wholeLV_(*t*) and using the same weighted nonlinear least squares minimization with a penalty for negative *C*_*b*_(*t*) and *C*_*t*_(*t*) values and residual blood activity in late time frames (last 4 minutes) [5]. Based on this estimate of *β*, (6) provided a new TAC for *C*_*b*_(*t*) that, once substituted as the blood input function *C*_blood_(*t*) in (1) and (2), allowed for regional estimates of MBF for each polar map sector.

#### 2.4.3. Arterial Input ROI

Two blood regions were derived, from which the arterial input function, *C*_blood_(*t*), could be sampled (average of all pixel values within the ROI).

**Figure 2:**
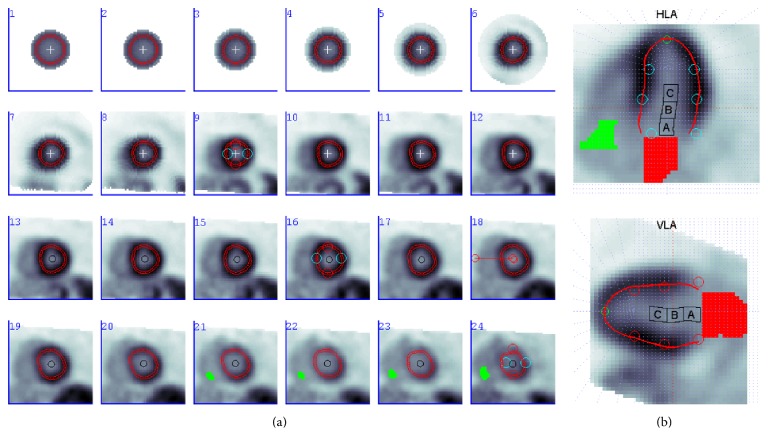
FlowQuant sampling of LV myocardium and arterial blood regions. On the left (from left to right, top to bottom), there are short-axis (SA) planes, which are consecutive slices through the horizontal and vertical long axis (HLA and VLA, resp.), shown on the right. The RA cavity pixels are shown in green, and the LA cavity pixels are shown in red. Late uptake frame derived LV blood-pool regions are shown as A (atrium), B (base), and C (cavity) regions in the HLA and VLA and as circles in the SA slices.

*Uptake-ROI.* Three (atrium, base, and cavity) warped cylinders (8 mm diameter) were positioned in the LV blood cavity so as to maximize their distance from the myocardial wall in each short-axis plane. Each region was sampled individually and the median intensity at each time frame was used to generate the blood input function *C*_blood-uptake-ROI_(*t*) [11]. The three regions are shown as A (atrium), B (base), and C (cavity) in Figure 2.

*Blood-ROI.* The blood-pool frame was determined using the point of maximum activity in *C*_blood-uptake-ROI_(*t*). The early blood-pool image was then masked to include only regions beyond the LV mitral valve plane (i.e., atrium and aorta) on which a threshold (85% of maximum) was applied. This region was then sampled to generate the blood input function *C*_blood-ROI_(*t*) [5], shown as the red patch in Figure 2.

#### 2.4.4. Right Blood Correction (RBC)

RV blood contamination of the sampled LV myocardium TAC is expected, particularly in the septal wall region. Thus, the following revised model accounting for RV blood spillover was derived previously [14] and investigated [17]:

(7) Cmyot=LVBV×Cbloodt+RVBV×Crvt+1−LVBV−RVBV×Ctt,

where LVBV is the left ventricle blood fraction, RVBV is the right ventricle blood fraction, and *C*_rv_(*t*) is the TAC in the right ventricle blood pool. To sample *C*_rv_(*t*), an RV blood ROI was derived on the frame preceding (10 sec) the peak LV blood, using a mask extending radially beyond the septal wall and applying a threshold at 85% of the maximum blood activity, as shown in green in Figure 2. RBC was implemented by substituting (3) with (7) in the septal half of the LV polar map and thus could be utilized with or without SOC, with any kinetic model, and with either Blood- or Uptake-ROI.

#### 2.4.5. Delay between Left Ventricle Cavity and Myocardium

A final modification to the standard model accounted for blood transport* delay* between left chambers of the heart and the perfused myocardium as follows:

(8) $$\begin{array}{c} C_{t}\left(\;t\;\right)=\left\{\begin{array}{cc} K_{1}e^{-\left(K_{1}/DV\right)\left(t-delay\right)}⊗C_{blood}\left(t\right), & t>delay\\ 0 & t\leq delay. \end{array}\right. \end{array}$$

To our knowledge, this transport delay has not been previously modeled in the context of cardiac PET. A similar delay is used in cardiac CT where the temporal resolution is much higher (~1 sec) [18].

### 2.5. Myocardial Blood Flow Analysis

All combinations of the model variants (2^5^ = 32 in total) were evaluated. Average rest and stress MBF, MFR, and ΔMBF were measured in the three coronary artery territories: left anterior descending artery (LAD), left circumflex artery (LCX), and right coronary artery (RCA), according to the American Heart Association guidelines [19]. These are reported throughout this work.

Using (4), the flow values using SOC-On Global-DV were significantly reduced compared to SOC-Off Global-DV and all of the Regional-DV values, which were previously calibrated to ^13^N-ammonia flow values [15]. In order to preserve MBF accuracy, a separate calibration (extraction correction) function was derived to correct for this bias by minimizing the mean squared error between the SOC-On Global-DV and the rest of the MBF values. The resulting extraction function for SOC-On was determined to be

(9) $$\begin{array}{c} K_{1}=\left(\;1-{0.76e}^{-0.40/MBF}\;\right)MBF. \end{array}$$

### 2.6. Data Quality Assurance

As part of routine quality assurance, a gamma-variate function was fitted to the blood TACs of each scan to ensure consistency of the blood TACs between test-retest scans, with typical profiles. The fitted parameters were used to resolve the following metrics: rise time (*t*_rise_), clearance time (*t*_clearance_), and mean transit time (*t*_mean-transit_) [20]. Scans with outlying gamma-variate parameters were excluded from further analysis, due to inconsistent tracer injection profiles.

### 2.7. Statistical Analysis

Continuous and discrete data are presented as mean ± standard deviation [minimum, maximum]. Test-retest analysis was performed using Spearman correlation (*ρ*). Additionally, Bland-Altman analysis was performed. Nonparametric tests were used to account for outliers and non-Gaussian distribution of the data. Differences in repeat flow measurements were calculated both in absolute terms and in relative percentage to the means of test and retest. For normally distributed difference data, absolute and relative repeatability coefficients (RPC = 1.96*∗*standard  deviation) are typically used to summarize the data. However, we noted that the flow differences data do not follow a normal distribution so nonparametric repeatability coefficients (RPC_np_) were presented as a measure of data variability (RPC_np_ = 1.45 × interquartile  range − IQR). For normally distributed data, RPC and RPC_np_ are equivalent [21, 22]. In order to account for systemic biases, median rest and stress MBF differences were subtracted, respectively, to force the median MBF differences to zero. We evaluated whether differences in rate pressure product (RPP) correlated with differences in flow. Wilcoxon and Levene's tests were used to test the statistical significance of differences in medians and variances, respectively [23]. *p* values < 0.05 were considered statistically significant. All the analysis was performed in Matlab R2013b.

## 3. Results

### 3.1. Patient Demographics

Twelve participants were recruited for this study, including 2 healthy volunteers and 10 patients with known or suspected CAD referred for clinical diagnostic testing. The demographic and hemodynamic data of these patients are detailed in Tables 1 and 2, respectively.

No RPP adjustments of rest or stress MBF values were performed, since the test-retest changes in MBF (delta) were not significantly correlated with changes in RPP (*R* < 0.30; *p* = NS). As expected, retest versus test RPP values were highly correlated (*R* ≥ 0.90; *p* < 0.001). A small but statistically significant increase in retest MBF was registered at stress (Table 3).

### 3.2. Patient Hemodynamics

Subject hemodynamic measurements are summarized in Table 2. No significant differences were found between test and retest for either heart rate or blood pressure at rest or stress, confirming stable hemodynamics. Rate pressure products (RPP) were significantly higher at stress compared to rest, confirming hemodynamic response to the stressor. Based on these population averages, rest and stress MBF values were normalized by multiplying by the population average RPP (8240 and 10638 bpm × mmHg for rest and stress, resp.) and dividing by the study specific RPP to reduce RPP related variability in MBF. However, this did not improve the results and in fact it made them worse, likely due to the propagation of RPP error (data not shown); thus, only non-RPP-adjusted values are reported.

### 3.3. Flow, Flow Reserve, and Flow Delta Values

**Figure 3:**
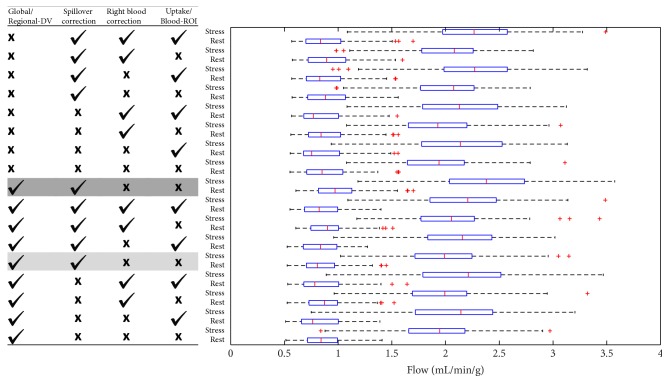
Myocardial blood flow values (for all 3 coronary territories combined), using each method. The method with the best test-retest repeatability was selected as the reference method to which other methods were subsequently compared (grey highlight). Delay-On (dark grey) is presented only as a variant of the reference method, for comparison.

Rest and stress MBF values are summarized in Figure 3 using boxplots. As expected, all methods showed significantly higher flow values at stress versus at rest (*p* < 0.0001), indicating effective response of the patients to the stressor and the ability of MBF to distinguish between physiologic states. The model variant with Blood-ROI, SOC-On, RBC-Off, Delay-Off, and Global-DV has been characterized previously [9] and was therefore selected as a reference method to which the other methods were compared. *K*_1_, MBF, and RPP-adjusted MBF values for this model are summarized in Table 3.

**Figure 4:**
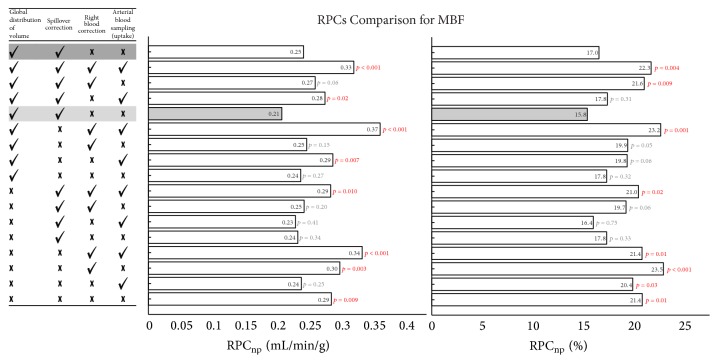
Absolute and relative MBF nonparametric repeatability coefficients for all methods with *p* values of comparison to references method (highlighted in grey). The single Delay-On variant is indicated with dark grey.

The transport Delay-On model was determined to have poor convergence and therefore was only reported with SOC-On, RBC-Off, Blood-ROI, and Global-DV. Detailed results for this model and the remaining 16 model variants are listed in Figure 4.

No significant differences in mean MBF values were measured between the model variants (RBC-Off versus RBC-On, Blood-ROI versus Uptake-ROI, and Global-DV versus Regional-DV) at either rest or stress using Wilcoxon analysis, which indicates no biases between methods. Likewise, no bias was measured for SOC-On versus SOC-Off due to calibration of the extraction correction function using the same dataset, as described.

### 3.4. Test-Retest Repeatability of Kinetic Model Variants

**Figure 5:**
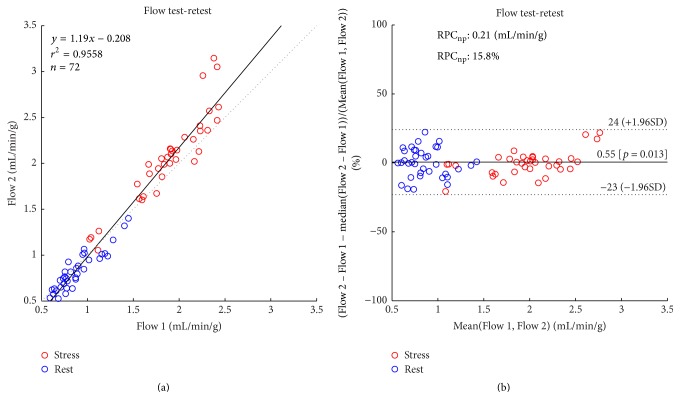
Correlation (a) and relative Bland-Altman (b) analyses of test-retest flow (rest and stress in 3 coronary territories) values using the reference method. Relative differences were expressed as a percentage of mean values, to account for increased variance at higher flow rates.

Repeatability coefficients (absolute and relative RPC_np_) are presented in Figure 4 for the various methods. The most repeatable method (with the lowest RPC_np_ values) is emphasized and corresponds to the reference method. The corresponding correlation and relative Bland-Altman analysis of MBF values are demonstrated in Figure 5 for the reference method.

**Figure 6:**
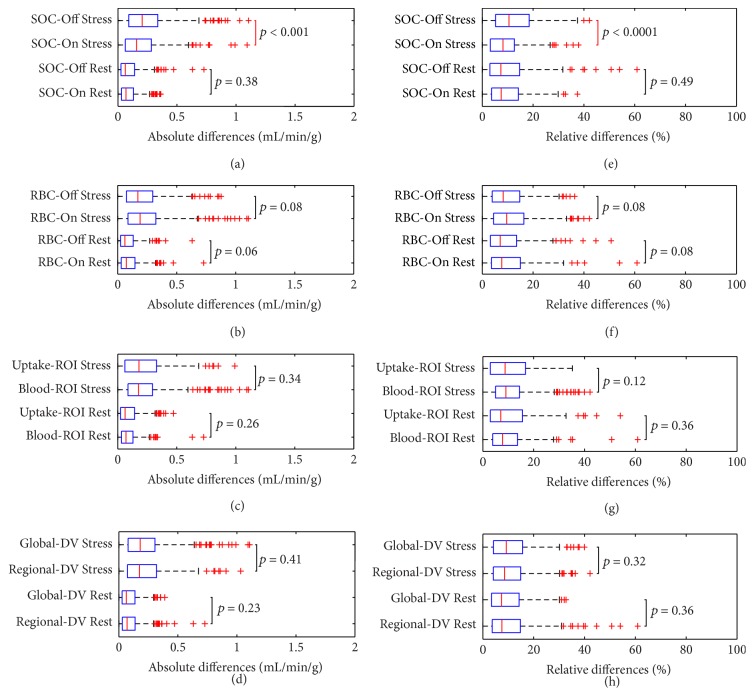
MBF: absolute test-retest flow differences (a, b, c, d) and relative flow differences (e, f, g, h) between model variants for rest and stress flow values using SOC-On versus SOC-Off (a and e), RBC-On versus RBC-Off (b and f), Uptake-ROI versus Blood-ROI (c and g), and Global-DV versus Regional-DV (d and h).

The individual effects of the model parameters on test-retest repeatability are shown in Figure 6. SOC-On had significantly lower test-retest flow differences than SOC-Off (*p* < 0.001 at stress). RBC-Off tended to be more repeatable than RBC-On (*p* < 0.08) but did not reach significance. Global-DV and blood input ROI model variants did not result in a significant difference in test-retest flow differences. Uptake-ROI was as repeatable as Blood-ROI, regardless of SOC-On or SOC-Off settings. Similar findings were observed for MFR and ΔMBF, with reduced MFR variability using SOC-On versus SOC-Off (*p* < 0.0001) and reduced ΔMBF variability using SOC-On versus SOC-Off (*p* < 0.0001) with an additional significant difference for Blood-ROI versus Uptake-ROI (*p* < 0.05).

### 3.5. Nonconvergence of the Delay Method

In the delay model, the transport delay parameter failed to converge in 57% of all sectors (*n* = 496 × 13 × 4), resulting in lower or upper boundary limits (0 or 10 s, resp.). Sample FlowQuant reports with Delay-Off and Delay-On are demonstrated in the Supplemental Material, SM 1 and SM 2, available online at https://doi.org/10.1155/2017/6810626. In comparison, for all the Delay-Off model variants, nonconvergence was observed in only 0.3% of segments.

## 4. Discussion

In this work, we evaluated the test-retest repeatability of ^82^Rb PET MBF, MFR, and ΔMBF values using several variants of the 1-tissue-compartment kinetic model analysis. While other kinetic models do exist, we focused on this particular model as it is the most widely accepted in the community [3] and comparison between our reference method and the simplified retention model was previously reported [6].

Our results suggest that the test-retest variability is the lowest using the left atrium Blood-ROI, SOC-On, RBC-Off, Delay-Off, and Global-DV. This conclusion is consistent with our previous work [5] using rest data alone in a separate patient cohort and the same software analysis program. The novelty of the current study is that it additionally evaluates the use of RV blood spillover correction, arterial blood transport delay, and a regional DV estimation. Furthermore, this study adds stress MBF, MFR, and ΔMBF repeatability measurements, which are pertinent to clinical utility. The present work reinforces our earlier conclusions that spillover correction, left atrium blood-pool derived input function, and the use of a global DV constant are preferable for repeatable MBF measurements using ^82^Rb PET.

In this work, we also determined that, with SOC-On and Global-DV, *K*_1_ values were lower compared to those of SOC-Off and Global-DV, consistent with a higher blood peak of the spillover-corrected arterial blood TACs, as previously observed using factor analysis for spillover correction [24]. SOC-Off MBF values were previously calibrated to ^13^N-ammonia-derived MBF values in a human population [15] and have been shown to agree with other software packages [3]. For this reason, SOC-Off Global-DV MBF values were used as a reference to which SOC-On Global-DV values were calibrated. Calibration to an external gold standard (e.g., ^13^N-ammonia or ^15^O-water PET) was beyond the scope of this work but may be warranted in future studies.

### 4.1. Comparison to Previous Literature

#### 4.1.1. Most Repeatable Method

To our knowledge, the lowest test-retest variability for ^82^Rb PET MBF values has been reported on a dataset that consisted of rest scans alone [5]. The rest-only repeatability coefficient in the present study was 0.19 mL/min/g or 21% relative to the mean, which is not significantly different from those previously reported in a separate cohort, 0.2 mL/min/g, or 24% relative to the mean.

An outlier in stress flow values may be noted in Figure 5. Visual inspection revealed considerable motion on the test-stress scan. Upon manual motion correction of the reconstructed dynamic image sequence, the flow value was reduced from 2.9 mL/min/g to 2.4 mL/min/g which is closer to MBF value derived from the motion-free stress retest data of 1.9 mL/min/g. This suggests that patient motion can have a serious impact on MBF measurements and warrants further work to detect and correct patient motion. Excluding this patient from the cohort would maintain the stress-only variability from 0.25 mL/min/g or 14.0% to 0.25 mL/min/g or 14.8%, since RPC_np_ is robust to the presence of outliers. However, none of the data used in this work was motion corrected, as this was beyond the scope of this work and due to missing ground truth to validate such correction.

Previous literature, without the use of SOC, has demonstrated MBF variability with anatomical placement of the arterial blood ROI (e.g., LV cavity, LA cavity, and aorta) [25]. While Vasquez et al. reported a bias, we noted that SOC-On reduces the Blood-ROI (compared to Uptake-ROI) bias in stress (0.16 to 0.12 mL/min/g; *p* < 0.00001) and delta (0.20 to 0.16 mL/min/g; *p* = 0.001), but not in rest or reserve (*p* > 0.05). It is worth noting that Klein et al. used the simplified retention model, which does not account for the shape of the blood input function and therefor may be more susceptible to ROI variations [6].

We also found no significant difference in MBF values between RVB samplings at the atrium or ventricle. However, this data was not shown out of considerations for conciseness and clarity—thus we opted to present the right atrium blood sampled data in which myocardial tissue signal spillover effects are smaller.

Another important contribution to the precision values reported by our group is the use of constant-activity infusion of the tracer over a 30-second interval as opposed to a fast tracer bolus [6].

#### 4.1.2. Study Design and Sources of Variability

In Klein et al.'s study [11], we evaluated the operator-dependent variability in MBF values for the same image analysis software. Intraoperator variability (RPC) was in the range of 4 to 8%. Combined rest and stress MBF RPC was <25% in this work, and thus it may be argued (assuming noise adds in quadrature) that only ~5% of the measured test-retest variability is associated with operator variability and the majority is associated with physiologic changes in patient hemodynamics and measurement noise. Although the experiment was designed to achieve hemodynamic stability between test and retest, some variation is to be expected. Regardless, these results highlight the degree of uncertainty in MBF, MFR, and ΔMBF measurements that can be expected in a human population using ^82^Rb PET and current image analysis methods.

### 4.2. Nonconvergence of the Delay Method

The addition of a blood transport delay parameter to the model resulted in poor convergence. Work in other modalities, including microbubble contrast enhanced (MCE) echocardiography and contrast enhanced dynamic X-ray computed tomography, has clearly demonstrated that the transport delay is on the order of 1-2 cardiac cycles (i.e., 1-2 sec) [26]. In the current work, temporal resolution was limited to 10 sec time frames, which likely resulted in insufficient temporal resolution to adequately resolve the transit time.

Reconstruction of shorter time frames has previously been investigated [27], but at the cost of higher image noise due to lower count statistics. We were not able to evaluate the effect of shorter time frames on the delay enabled model as the list-mode data was not available post hoc. It is unclear whether the delayed model may be beneficial with faster temporal sampling in ^82^Rb PET.

### 4.3. ROI Size

Blood-ROI and Uptake-ROI sizes were 4.8 ± 0.6 mL versus 28.5 ± 7.8 mL, respectively. Thus, Blood-ROIs were smaller and more reproducible in size than Uptake-ROIs. Since the blood input function is an average of voxels within the ROI, the ROI size is secondary to the position of the ROI relative to anatomical structures. Nevertheless, ROI selection did not significantly impact MBF repeatability in this work.

### 4.4. Applications

This study quantifies the test-retest variability associated with the entire imaging pipeline from acquisition to kinetic modeling and is therefore important for quantifying the repeatability of MBF, MFR, and ΔMBF quantification using ^82^Rb PET imaging. In clinical applications, these estimates are important for establishing confidence intervals when comparing individual scans to normal reference database values and for evaluation of serial changes in response to disease progression and/or therapy. In a research setting, these estimates are essential for planning sample sizes to detect significant experimental effects.

### 4.5. Study Limitations

The primary limitation of this work is the relatively small number of patients (*n* = 12). Nevertheless, we were able to demonstrate statistically significant RPC differences between several model variants.

While the results of this work demonstrate the superiority of the reference method for test-retest repeatability, the absolute and relative RPC values disclosed in the work are only applicable to our specific patient population, constant-activity rubidium infusion, PET scanner, image reconstruction protocol, image analysis methods, and software. Repeatability measurements may differ depending on these and perhaps other factors, such as image binning protocols, image segmentation and sampling, time-frame weighting, and extraction correction. However, these factors are not the focus of the work presented herein.

The stress test and retest measurements were not performed under identical hemodynamic conditions due to administration of dipyridamole and aminophylline relative to times of acquisition. However, there were no significant differences in hemodynamic parameters (heart rate or blood pressure) or in stress MBF values between test and retest. Furthermore, any differences in the experimental conditions would likely result in increased variability between measurements, and as such the reported repeatability measures serve as an upper limit of the true single-stress test-retest variability.

Finally, while this work did not directly evaluate the accuracy of MBF measurements against a gold standard, we adjusted for known MBF biases in the SOC-On method using a surrogate method (i.e., SOC-Off), which was previously calibrated for accuracy using ^13^N-ammonia PET in healthy normal subjects and CAD patients [22].

## 5. Conclusions

Amongst several alternative 1-compartment model variants, MBF, MFR, and ΔMBF repeatability was the best using dual myocardium blood spillover correction and using a global DV constant, while RV blood spillover correction and arterial blood sampling ROI did not have a statistically significant influence on repeatability. Temporal sampling was insufficient to estimate blood transport delay.

The 95% limits of agreement for MBF values were less than 25% of the mean at rest and stress, which may be used clinically to determine confidence intervals for detecting serial changes and comparison to population databases and also to determine sample sizes in research studies, using ^82^Rb PET MBF.

## Supplementary Material

In Supplementary Material we present (i) a sample FlowQuant report for clarification on how the software is used and (ii) detailed explanation of how the above mentioned kinetic model's report is actually being interpreted. The first section demonstrates a converged analysis, and the second one demostrates the lack of convergence of the Delay-On kinetic model variant.

## Figures and Tables

**Table 1:** Patient demographics (*n* = 12).

Healthy volunteers/CAD patients	2/10
Sex (females/males)	5/7
Age (mean ± SD [range])	61.1 ± 11 [46–81] years
BMI (mean ± SD [range])	32.9 ± 5.9 [24–43] m^2^/kg
Diabetic (no/IDDM/NIDDM)	10/1/1
Smoker (never/current/past > 1 yr)	6/3/3

BMI: body mass index.

IDDM: insulin-dependent diabetes mellitus (type 1).

NIDDM: non-insulin-dependent diabetes mellitus (type 2).

**Table 2:** Patient hemodynamics (*n* = 12).

Parameter	Rest 1	Rest 2	Stress 1	Stress 2
HR (bpm)^*∗*^	65 ± 8	65 ± 8	84 ± 13	83 ± 11
Systolic BP (mmHg)	124 ± 11	123 ± 10	129 ± 19	123 ± 16
Diastolic BP (mmHg)	73 ± 8	73 ± 6	76 ± 13	70 ± 11
HR × sys. BP (bpm × mmHg)^*∗*^	8092 ± 1242	8083 ± 1301	10869 ± 2549	10197 ± 2169

^**∗**^ *p* < 0.05 for all rest versus stress.

*p* = ns for Rest 1 versus Rest 2 and Stress 1 versus Stress 2 for all parameters.

**Table 3:** Uptake (*K*_1_), flow (MBF), TBV (total blood volume), and *β* (fraction of pure blood signal) values for reference method.

Parameter	Rest 1	Rest 2	Stress 1	Stress 2
*K*_1_^*∗*^ [mL/min/g]	0.51 ± 0.08	0.50 ± 0.08	0.82 ± 0.12^†^	0.87 ± 0.15
MBF [mL/min/g]^*∗*^	0.91 ± 0.25	0.88 ± 0.22	1.99 ± 0.45^†^	2.19 ± 0.45
MBF (RPP-adjusted)^*∗*^ [mL/min/g]	0.91 ± 0.23	0.89 ± 0.21	1.99 ± 0.51^†^	2.33 ± 0.62
TBV	0.28 ± 0.05	0.27 ± 0.05	0.31 ± 0.05^†^	0.30 ± 0.04
*β*	0.81 ± 0.05	0.79 ± 0.03	0.86 ± 0.04^†^	0.85 ± 0.03

^*∗*^ *p* < 0.05 for all rest versus stress.

^†^ *p* < 0.05 between Rest 1 and Rest 2 and between Stress 1 and Stress 2.
