# Supplementary material for: Optimally Repeatable Kinetic Model Variant for Myocardial Blood Flow Measurements with 82Rb PET
Source: Comput Math Methods Med. 2017 Feb 13;2017:6810626. doi: 10.1155/2017/6810626 (PMC5331165; doi:10.1155/2017/6810626)
Supplement: Supplementary file 1 — In Supplementary Material we present (i) a sample FlowQuant report for clarification on how the software is used and (ii) detailed explanation of how the above mentioned kinetic model's report is actually being interpreted. The first section demonstrates a converged analysis, and the second one demostrates the lack of convergence of the Delay-On kinetic model variant. [file 6810626.f1.docx]

# Supplemental Material

## Sample FlowQuant Reports

A typical rubidium PET MBF analysis report is shown in SM 1. The dynamic scan data is oriented and aligned to the LV long-axis using a semi-elliptical shape fit algorithm (shown in the left top part of the image). Then the myocardial ROIs are positioned to sample the LV activity. The arterial blood input is sampled in the area highlighted with the red pixels or in the ABC (Atrium, Base, and Cavity) regions (shown in the top right of the image).

The ‘high uptake’ sampled TACs are shown in the bottom left part of the image.

For all the 496 sampled sectors of the myocardium, individual *K_1_* and FBV parameters are fitted to estimate the regional flow values.


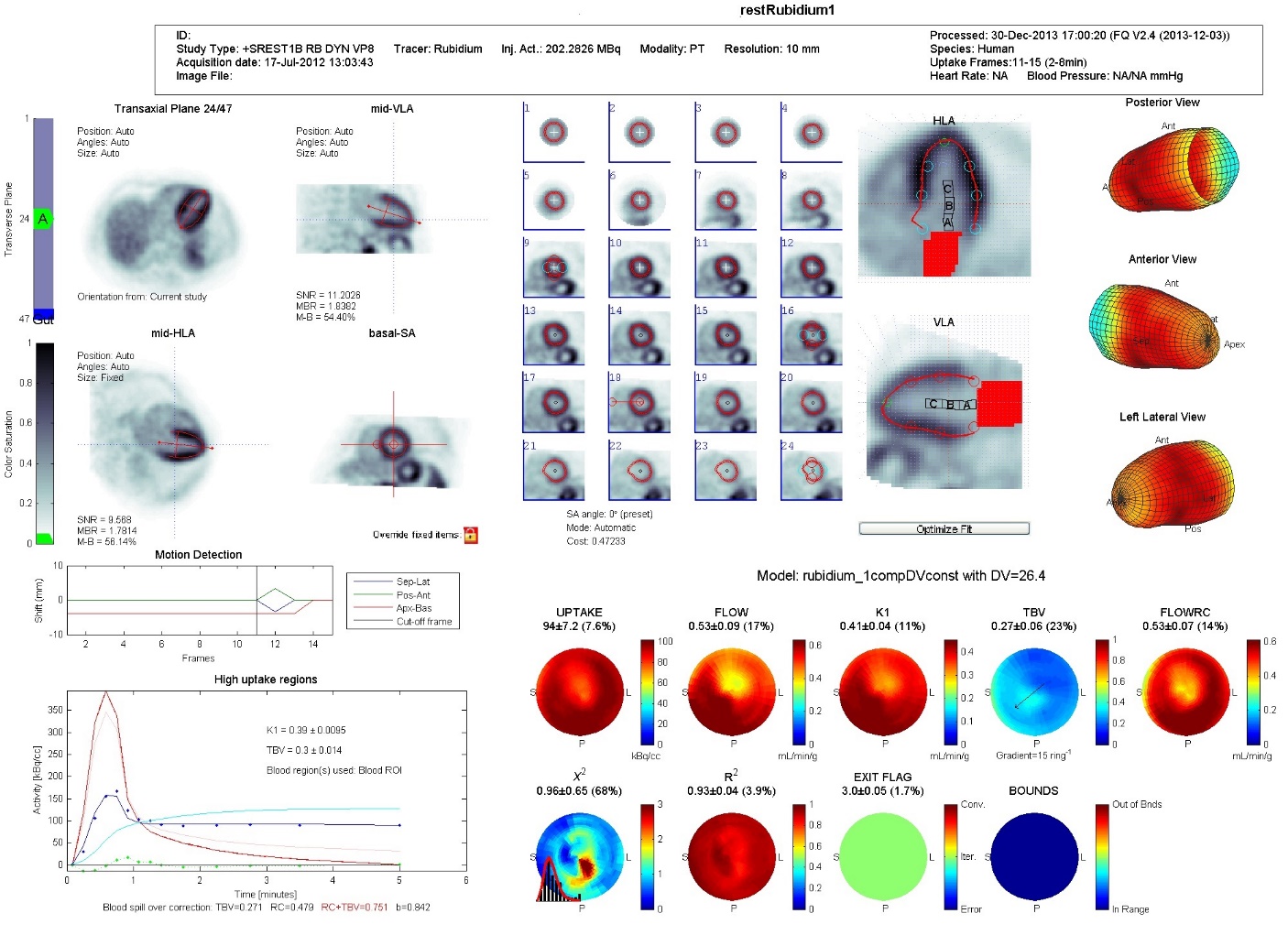


SM 1 - Typical FlowQuant® kinetic modeling report for a rest ^82^Rb dynamic scan for quantification of MBF.

## Kinetic Modeling Interpretation

The kinetic modeling portion of the report includes modeling and quality of fit parameters, in a series of polar map representations. The example in SM 1 shows good convergence of the kinetic model with no parameters at boundary limits. In SM 2 the kinetic modeling report for the same data as SM 1 is show, but with the blood transit delay model enabled. A large portion of the Delay polar map in this example is at the upper or lower limits (1 or 10 seconds respectively), as is also indicated in red on the Bounds polar map, indicating poor convergence of the model.


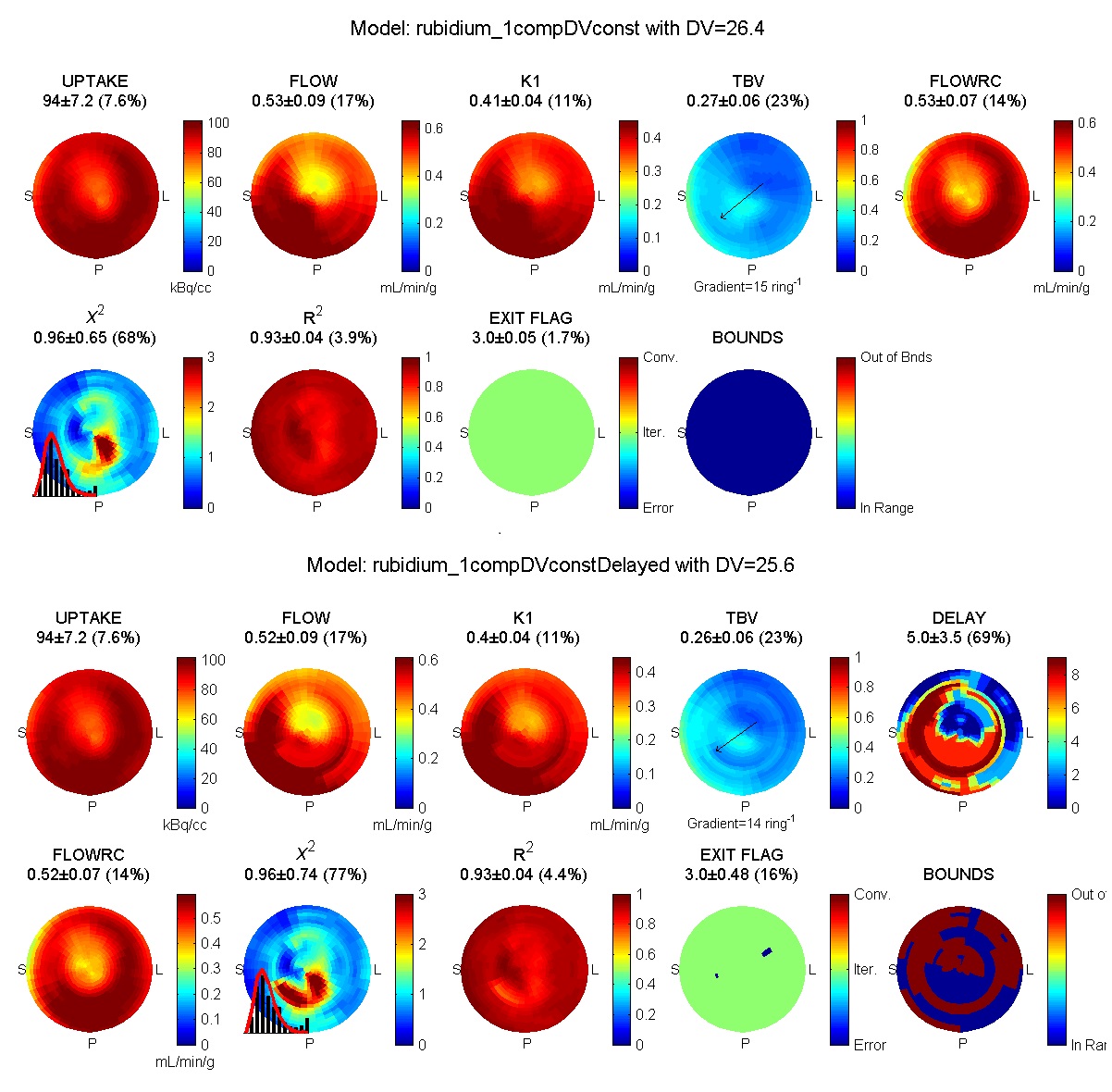


SM 2 – Kinetic model summary results for the same data as in SM 1 with modeled blood transit delay model included. Note the differences between the BOUNDS polar maps, indicating multiple sectors are at boundary limits (red) on the bottom image, corresponding to the Delay parameter polar-map.
